# Dietary strategies and food practices of pediatric patients, and their parents, living with inflammatory bowel disease: a qualitative interview study

**DOI:** 10.1080/17482631.2019.1648945

**Published:** 2019-08-06

**Authors:** Kim H. Chuong, Jennie Haw, Alain Stintzi, David R. Mack, Kieran C. O’Doherty

**Affiliations:** a Department of Psychology, University of Guelph, Guelph, ON, Canada; b Ottawa Institute of Systems Biology and Department of Biochemistry, Microbiology and Immunology, Faculty of Medicine, University of Ottawa, Ottawa, ON, Canada; c Children’s Hospital of Eastern Ontario (CHEO) IBD Centre and Department of Pediatrics, University of Ottawa, Ottawa, ON, Canada

**Keywords:** Children, chronic illness, diet, healthy eating, inflammatory bowel disease, nutrition, thematic analysis

## Abstract

**Purpose**: A growing body of scientific evidence supports the role of food and diet in the pathogenesis and management of inflammatory bowel diseases (IBD). However, little is known about the role of food and diet from the perspectives of pediatric patients and their parents. This study aimed to explore how children and adolescents with IBD and their parents coped with the illness through food and diet in their daily lives.

**Methods**: We conducted semi-structured interviews with 28 children and adolescents with IBD, 26 parents and one grandparent.

**Results**: Two major themes, dietary strategies and family food practices, were identified through thematic analysis. There were three types of dietary strategies: food avoidance and moderation; following a specific diet; and healthy eating. For family food practices, two subthemes were identified: impact on grocery shopping, meal planning, and cooking; and maintaining routine and normality.

**Conclusions**: Our findings have important implications for the clinical care of pediatric IBD. Notably, IBD not only influenced the food practices of the pediatric patients, but also their parents and other family members. Healthcare professionals should consider the family unit when giving nutritional advice or developing nutritional guidelines. Personalized nutritional counselling and ongoing nutritional assessment are also warranted.

Inflammatory bowel diseases (IBD) are disorders of the gastrointestinal tract characterized by chronic inflammation of the intestinal mucosa. The two main subtypes are ulcerative colitis (UC) and Crohn’s disease (CD). IBD is emerging as a global disease with increasing incidence and prevalence rates in different regions around the world (Ng et al., 2017). The prevalence and incidence rates are highest in more developed nations, especially in North America and Europe (Ng et al., 2017). Individuals with IBD may experience periods of relapse and remission. Physical symptoms include abdominal pain, diarrhea, rectal bleeding, frequency and urgency, and systemic symptoms include fatigue, loss of appetite and weight loss. Furthermore, IBD is associated with nutritional deficiencies because of dietary restriction, increased nutritional requirements due to systemic inflammation, malabsorption of nutrients, medication and surgery (Kleinman et al., 2004; Lucendo & De Rezende, 2009; Massironi et al., 2013). Growth impairment is common in children and adolescents with CD, although less frequently observed in those with UC (Kleinman et al., 2004).

Currently, treatments are primarily directed to heal inflammatory responses to induce and maintain remission (Charlebois, Rosenfeld, & Bressler, 2016). Nutrition as therapy in CD has been recommended for children and adolescents to induce remission (Ruemmele et al., 2014), and nutritional guidelines for pediatric IBD have been recently published in a position paper on behalf of the Porto IBD group of the European Society of Pediatric Gastroenterology, Hepatology and Nutrition (ESPGHAN) (Miele et al., 2018). The guidelines were developed by a group of 20 experts in pediatric IBD who participated in an iterative consensus process, following an open call to the Pediatric IBD Porto Group, IBD Interest Group, and Nutrition Committee of ESPGHAN. This study aimed to explore the food practices of children and adolescents with IBD and their parents, specifically on how they used diet to cope with the illness in daily life and their perspectives regarding its impact on family food practices. By exploring the food practices of pediatric patients and their parents, this study provides insights to healthcare providers, dieticians, and researchers when giving nutritional advice or developing nutritional guidelines.

## The role of food and diet

The etiology of IBD is thought to result from a complex interplay of genetic susceptibility, the intestinal microbiome, and aberrant immunological reactions interacting with environmental factors. Among the environmental factors, diet has been considered to be the most likely to be modifiable; however what aspects of diet that are of importance are largely unknown (Lee et al., 2015). There is a growing body of scientific evidence associating the effects of diet on the intestinal microbiota and risk of IBD (Kostic, Xavier, & Gevers, 2014; Lee et al., 2015; Lewis et al., 2015, 2015; Winglee & Fodor, 2015). Amre et al. (2007) reported that imbalances in the consumption of fatty acids, fruits and vegetables increase the risk of CD among children. High levels of consumption of refined sugars and carbohydrates, red meats, and omega-6 polyunsaturated fatty acids have been implicated as risk factors (Lee et al., 2015; Lucendo & De Rezende, 2009), and restrictions of sugar and carbohydrate consumption have been popular notions. However, a recent report detailing the lack of mucosal healing with the specific carbohydrate diet (SCD), which eliminates most carbohydrates and sugars except for monosaccharides, has raised questions about its clinical utility (Wahbeh, Ward, Lee, Giefer, & Suskind, 2017), and the SCD is not recommended for pediatric IBD patients (Miele et al., 2018). In addition, supplementation with omega-3 free fatty acids was reported as not effective for the prevention of relapse in CD (Feagan et al., 2008; Lev-Tzion, Griffiths, Leder, & Turner, 2014). More recently, a prospective, multicenter study of patients with UC in remission on aminosalicylate therapy revealed that increasing intake of myristic acid (commonly found in palm oil, coconut oil, and dairy fats) was associated with disease relapse (Barnes, Nestor, Onyewadume, de Silva, & Korzenik, 2017). Other foods, such as processed meat, alcohol, and foods high in sulfur, were not associated with an increased risk of flare (Barnes et al., 2017).

Exclusive enteral nutrition (EEN) has been studied and shown to reduce inflammation and induce remission of CD (Lee et al., 2015). EEN involves taking a formula-defined liquid diet at the exclusion of usual dietary items. It is recommended as a first-line therapy to induce remission in pediatric CD by an international working group from ESPGHAN and the European Crohn’s and Colitis Organization (ECCO) (Ruemmele et al., 2014). EEN has good efficacy in inducing remission and fewer adverse effects than corticosteroids, although there are no specific guidelines on the choice of enteral formula as all seem to be equally efficacious (Zachos, Tondeur, & Griffiths, 2007). According to a review by Richmond and Rhodes (2013), EEN is effective for CD although many patients relapse within 6 months after their return to a normal diet. Although further evidence is needed, the Pediatric IBD Porto Group from ESPGHAN recommends avoiding a diet with high fat, high protein, high sugar, and low in fruit and vegetables as several epidemiological studies have found a positive correlation with the risk of developing IBD (Miele et al., 2018). Avoidance of foods with large amounts of emulsifiers has also been suggested (Miele et al., 2018). However, a diet low in fermentable oligo-, di- and monosaccharides and polyols (FODMAPs) is not recommended for induction or remission in children and adolescents due to the risks of nutrient deficiencies of such a diet over long term (Miele et al., 2018). Clearly, further studies about food consumption and IBD are still needed.

Research that investigates patient perspectives with regard to diet has largely focused on adults or young adults. Quantitative studies reported that many adult patients perceived food and diet as important in affecting their condition (Bergeron, Bouin, D’Aoust, Lemoyne, & Presse, 2018; Holt, Strauss, & Moore, 2017; Jowett et al., 2004; Kinsey & Burden, 2016; Prince, Whelan, Moosa, Lomer, & Reidlinger, 2011; Zallot, Quillot, & Chevaux, 2013; Zutshi, Hull, & Hammel, 2007). These studies reported that patients often avoided trigger foods that they identified as provoking their symptoms. Notably, the perceived importance of food did not differ significantly between patients in remission and those in relapse (Prince et al., 2011). Similarly, in qualitative interview studies, adult patients talked about the importance of food and identification of trigger foods for their condition, as well as challenges in restricting the consumption of those foods (Fletcher & Schneider, 2006; Palant et al., 2015; Schneider, Jamieson, & Fletcher, 2009). To our knowledge, there is no research that explores the role of food and diet from the perspectives of children and adolescents with IBD. The few existing studies have focused on opinions regarding specific dietary interventions. For instance, Svolos et al. (2017) reported that pediatric CD patients and their parents rated EEN as more difficult to adhere to when compared to a proposed exclusion solid food-based diet, even though most participants were positive about completing another EEN course during a relapse.

## The present study

This study is part of a larger project exploring the associations between IBD, diet, intestinal microbiota, and food environments. Given the paucity of research on the food experiences of pediatric IBD, we aimed to explore how children and adolescents with IBD and their parents coped with the illness through food and diet in their daily lives. Our analysis was guided by the following research questions: What are the food practices of pediatric patients and parents living with IBD? How does IBD influence their food practices? What food strategies, if any, do they use to manage IBD?

## Methods

### Design

We conducted a qualitative research study with an explorative design and an inductive thematic analytical approach. Thematic analysis provides a systematic method for identifying, analyzing, and reporting patterns across the dataset. It provides a theoretically flexible approach that can be applied across a multitude of disciplines, and is useful for applied research in health (Braun & Clarke, 2014).

### Participants

Participants were 28 children and adolescents, 26 parents and one grandparent. The children and adolescents were patients attending an IBD outpatient clinic located within The Children’s Hospital of Eastern Ontario (CHEO) in Ontario, Canada. At the CHEO IBD Centre, patients have consultation with a Dietician associated with the clinic at the time of diagnosis, by physician dictate and with patient request. The clinic research coordinator approached potential participants and their accompanying parent(s) to participate in the study. Purposive sampling was used to achieve sufficient data richness by seeking to maximize variation in terms of age, gender, ethnicity, and geographical location (i.e., living in urban or rural area). This was done in order to ensure that we captured a diverse range of perspectives and experiences among patients who visited the clinic during the recruitment period. In total, there were 12 girls and 16 boys aged 9 to 17 who participated in the study (Table I). Five had a diagnosis of ulcerative colitis (UC) and 23 had Crohn’s disease (CD). The length of time from diagnosis to interview ranged from 4 months to 11 years. None of the children and adolescents were on a specific dietary intervention provided by a healthcare professional at the time of the interview, and the majority were in remission. Participants received verbal and written information about the study and that their personal information would be kept confidential. Participants were also informed that they could decline to discuss any issues that they would prefer not to talk about and withdraw from the study at any time without consequences. All of the children and adolescents provided assent and parents provided consent to participate. Ethics approval was obtained from the Research Ethics Boards at The Children’s Hospital of Eastern Ontario (REB protocol no. 16/108X) and the University of Guelph (REB16-12–884).

**Table I.** Participant characteristics (n = 28).

Gender	
Female Male	12 16
**Age**	
Range	9 − 17 years
Mean ± SD	14 ± 2.14 years
**Condition**	
Crohn’s	23
Ulcerative Colitis	5
**Remission**	
Yes	22
No	6

*Note*. One participant withdrew from the study after giving consent and the interview was not conducted (P17).

### Data collection

The interviewer (JH) conducted semi-structured interviews with participants in April and May 2017 either before or after their appointment at the clinic, or arranged to conduct an interview by phone. The use of semi-structured interviews allowed us to explore particular issues that might arise during the interview in further depth than survey methods. An interview guide was developed to help guide the conversations with participants, but participants were free to raise new issues and share what was pertinent and meaningful to their experiences. The interview guide was used to probe participants about their household food practices, their local food environments, the meanings of food and meals for them and their family, and their knowledge of the relationship between food and IBD (See Table II for sample interview questions). The children and adolescents were interviewed separately from their parents unless they chose to be interviewed together. In four of the interviews, the child was interviewed together with a parent. In one interview, both parents were present. Interviews lasted from 10 to 60 minutes. Typically, interviews were longer if both children and parents participated together, and interviews with older adolescents tended to be longer than those with younger children.

**Table II.** Sample interview questions.

Topics	Sample Questions
Family food practices	Can you describe a typical family meal? Who does the meal planning, grocery shopping and food preparation in your family? What do you consider when you’re deciding whether or not to eat something/make a particular meal?
IBD and food	Can you tell me what you know about IBD and food? How does having IBD influence (or not) what you eat? (for children and adolescents) Can you describe any challenges in trying to include/avoid foods for IBD?
Food environment	What kinds of food options are available to you? (prompt for supermarkets, restaurants, school)
Food meanings and experiences	How would you describe the role that food and eating play in your family? What do meals mean to you? Mean to your family?

Interviews were audio-recorded and transcribed verbatim with participants’ permission. All interviews were conducted in English, with the exception of one parent interview that was partly in French. Translational help was provided by her adolescent son, who was interviewed at a separate time. The French sections of the interview were transcribed and translated into English.

### Data analysis

We analyzed the data according to the principles of thematic analysis (Braun & Clarke, 2006). Interview transcripts were uploaded into *NVivo10* to facilitate data storage and coding. Initial coding was done by the first and second authors (KC, JH), and a research assistant, according to a coding framework developed by the first and second authors. The coding framework was developed based on preliminary reviews of the transcripts, and new codes were added to the framework as coding progressed. Codes were checked by the first author across all interviews and were adjusted and collated to capture emerging patterns of meanings through an iterative process. During this process, the first author organized and refined recurring themes related to the research questions and potential links between themes into a draft analysis. The first author constantly revisited the interview transcripts and codes to ensure that themes reflected participants’ descriptions of their experiences. All co-authors reviewed and provided feedback on the analysis and interpretation of results. The children and adolescents were identified by participant number (e.g., P1), and parents were identified in relation to the participant number assigned to their children (M for mother and F for father plus the child’s participant number, for example, F1 is P1’s father).

## Results

Two major themes were identified from the interviews: dietary strategies and family food practices. We identified three types of dietary strategies that participant used: food avoidance and moderation; following a specific diet; and healthy eating. For family food practices, two subthemes were identified: impact of IBD on grocery shopping, meal planning, and cooking; and maintaining routine and normality for the family.

### Dietary strategies

#### Food avoidance and moderation

We found a range of experiences and strategies that participants used to manage IBD, with some relying more heavily on food and diet than others. While a few participants mentioned that they or their children did not have any trouble with food, the majority of participants talked about avoiding foods that they had found to provoke symptoms from past experiences. The types of foods that participants avoided varied widely, and included fast foods or fried foods, spicy foods, processed foods, gluten, and high-fiber foods such as raw vegetables. Others avoided more specific food items, such as corn and popcorn. For many participants, identification of foods that could trigger IBD symptoms was an important means by which to cope with the illness. One participant kept a food diary, suggested to her by the Dietician at the IBD Clinic, to keep track of foods that could aggravate her symptoms (P1, female, age 12). However, keeping a food diary seemed to be uncommon among the participants. Nevertheless, most participants relied on past experiences to help guide them on their food decisions. Several participants used the term, “trial and error,” to describe how they or their children had come to notice and avoid certain foods:

We tried to just, we did just like a trial and error kind of situation and so I was like, well let’s see if strawberries hurt my stomach, and then they didn’t, so I knew that I could eat strawberries. And then we tried not very much corn and we’re like, OK corn is a big no-no. (P9, female, age 15)

A few of the participants talked about listening to one’s body as a way to decide what foods to eat or not to eat. For several participants, how they dealt with food was a personal and individualized experience. One participant’s father said:

What I have found is that from listening to people tell me ‘oh it’s good if he eats this, it’s good if he eats that’ it’s—for me it’s whatever [P11, male, age 15] wants for his stomach. (F11)

Aside from avoiding trigger foods, many participants talked about moderating their intake of certain foods. One participant mentioned having trouble with fried food after she was diagnosed, but she could now consume it in moderate amounts:

I have to be very careful. I have to know the exact portion size of the fried food I’m getting because if it’s too much I’ll have problems. But certain sizes if I have, I’ll be fine. These days, now I can eat most trouble foods as long as I have a certain ratio of beverage with them, I will usually be fine. So, but if I just eat them by themselves though, it doesn’t end well. (P2, female, age 16)

Her comments illustrated how dietary patterns might change depending on if she was in remission or having active flare-up. Likewise, another participant indicated that she would check nutritional labels and moderate her intake of fiber during relapses:

I usually don’t eat, like when I’m on a relapse I usually try to stay away from things with skin and seeds, and I usually try to stick with anything under 3 grams of fibre. (P1, female, age 15)

Additionally, P1 mentioned that she would have small meals and Ensure (a supplemental nutrition drink) during relapses to avoid aggravating her symptoms. Eating smaller portions during relapses was also mentioned by other children and adolescents.

A few of the participants spoke of the difficulty in abstaining from trigger foods. For some participants, consuming foods that could trigger symptoms in moderate amounts allowed them to not completely eliminate those foods from their diet. For example, one participant’s mother (M21) said she would limit corn-on-the-cob to once a month in the summertime as “a special thing” because her son (P21, male, age 13) “really love[d] it.” Another participant (P5, female, age 14) identified grains and fibre as foods that could trigger her symptoms. Her mother (M5) indicated that the family had reduced rather than eliminated the consumption of pasta “because it was something that we were so accustomed to eating all the time.” Difficulty in abstaining from trigger foods had also been reported among adults and young adults with IBD (Fletcher & Schneider, 2006; Palant et al., 2015; Schneider et al., 2009).

#### Following a specific diet

Two of the participants were following the specific carbohydrate diet (SCD) at the time of the interview (P26, female, age 10; P28, female, age 12). This diet was initially developed in the mid-20^th^ century by gastroenterologist Sidney Haas to treat celiac disease and later popularized by Elaine Gottschall in the 1990s. The SCD eliminates grains and most carbohydrates and sugars except for monosaccharides, limits most dairy products, and recommends nut-based flours and homemade yogurt that is fully fermented and free of lactose. The SCD has been reported as resulting in clinical and mucosal improvements for children with IBD in several prospective (Cohen et al., 2014), and retrospective case studies (Obih et al., 2016; Suskind, Wahbeh, Gregory, Vendettuoli, & Christie, 2014). However, further research is needed to investigate the use of the SCD with larger and more diverse samples, to assess its safety and efficacy, and to understand the mechanisms underlying its effects (Cohen et al., 2014; Obih et al., 2016; Suskind et al., 2014). Moreover, the SCD is a very restrictive diet that can be difficult to follow strictly, and is not recommended for children and adolescents (Miele et al., 2018). According to Wahbeh et al. (2017), patients who follow the SCD may ease off the restrictions imposed by the diet after a period of following it and include some restricted foods in a modified SCD (e.g., rice, oats). However, their retrospective case study reported a lack of complete mucosal healing among children with CD after being treated exclusively with a modified SCD.

For P26 and P28, their parents had a large influence on the decision to follow the SCD. P26’s mother mentioned that she researched the diet based on a friend’s recommendation and had also adopted the diet herself in support of her daughter:

It just seemed, um, and I know, like, um, there’s no hard and fast scientific evidence behind it, but it seemed to make sense to me because prior to that, we were really struggling with, um, what to- what would help [P26] … it was something that I felt I could do for her and she really wants to have a cure and she wants to do something so she was right on board as well, so. She and I are both doing it together. (M26)

P28 was following the SCD for the second time when she was interviewed. She had previously adopted the SCD for six months and decided to start the diet again as a way to improve her health. She first followed the diet because her father had personal experience with it. Her father commented:

15 years ago I went into my difficulties with my bladder. I was not in good shape. I was in really bad shape. I tried all the drugs that they were doing for ulcerative colitis is what they said, um, but nothing was working. I was just taking pill after pill, whatever else they gave me. And I found a book called, *Breaking the Vicious Cycle*, by Elaine Gottschall … I did it completely for, I think it was about 8 months, I lost a boatload of weight, I was kinda gross by the end of it to be honest with you, but it worked. (F28)

Both P26 and P28 expressed difficulty in adhering to the SCD, but also a determination to follow through with it. P26 expressed that “it’s pretty daunting when I see the kids having like gummies in their lunches and noodles, so- but, yeah I can cope with it. I’m getting better with it.” Similarly, P28 described that “it’s very hard and I don’t like it that much, but that’s OK.”

#### Healthy eating

In addition to food avoidance and moderation, many participants emphasized healthy eating. Healthy eating is a strategy that is encouraged by the Dietician at the IBD Clinic. For the majority of these participants, healthy eating consisted of consuming more fruits and vegetables. Several participants spoke of healthy eating in relation to its positive effects on IBD. One participant (P18, female, age 16) stated: “I try to eat more fruits and veggies because apparently, like, the vitamins that’s in it is good for, it can be good for Crohn’s.” Another participant (P25, female, age 14) indicated: “If you eat healthy, there’s less of a chance that you can get worse symptoms.” Yogurt was also referred to as healthy and good for IBD by a few children and parents. One participant’s mother explained: “I try to get her [P2, female, age 16] to eat yogurt, you know, ‘cause of the probiotics.” (M2)

The concept of whole foods, or foods that have been processed or refined as little as possible and are free from additives or other artificial substances, is also introduced to patients and their families by the IBD Clinic Dietician. Many children and parents brought up the notion that foods that were fresh or considered natural (e.g., not processed, free of additives) were healthier and better for IBD. One participant’s mother (M21) indicated she would buy deli meats free of nitrates and avoid sausages because she heard that nitrates could aggravate IBD symptoms. A few parents mentioned grocery shopping for organic foods. This is exemplified in the interview with P25’s (female, age 14) father:

So organic’s a big thing, and I know there’s some debate about whether that’s better or not, but I tend to buy specific organic products that I know are really organic and they’re not just labeled. Um, I have a list of- I’ve compiled over the years, of- of- you know, basically doing research on the net saying ‘this stuff is actually legitimately-‘ you know, Farm Boy has a great line of organic products that they certify are organic and they’re all stamped with lots of things. Um, so I- I try to go organic as much as I can. (F25)

Other aspects of healthy eating included limiting foods that were perceived as unhealthy (e.g., foods high in sugar and salt, processed foods, fast foods), opting for food choices that were considered healthier, and balanced eating of different food groups and a variety of foods. For some participants, foods that they considered as unhealthy might also be those that could aggravate IBD symptoms (e.g., fast foods were often mentioned as both unhealthy foods and trigger foods). One participant commented that his family placed importance on healthy eating and he would opt for healthier food choices when eating out:

We sort of strive towards like healthier foods. Like if there was a choice between going to Wendy’s or McDonald’s versus going to Subway, we would usually go to Subway. (P12, male, age 15)

All three options are popular fast food restaurant chains in Ontario. However, P12 considered Subway a healthier option, likely because of its promotion as a healthier choice with fresh ingredients. As well, Subway allows its customers to choose which vegetables they want, which might have helped in its promotion. P12 referred to the consumption of fresh vegetables and fresh meats as important for healthy eating. In contrast, he related the consumption of unhealthy fast foods to both IBD symptoms and lower psychological well-being:

I just hate being sick so, and I just feel miserable, so usually like fast foods and stuff just make me like really lazy and sort of just disgusted, or I just feel mopey. So I sorta just stay away from them. (P12, male, age 15)

Balanced eating of different food groups and a variety of foods was discussed in relation to healthy eating directly or indirectly by several children and parents. For example, when describing how she planned for family meals, the mother of P5 (female, age 14) said:

I usually try to, I’ll make sure we have some kind of a meat and a vegetable and then I usually, we’ll have like a potato or some kind of, like, rice or pasta or something with it. So, yeah, I just try and include those 3 things in in each meal. (M5)

A few parents and children mentioned the need to ensure the inclusion of certain key nutrients in their meals, particularly proteins. For example, one participant’s father (F25) emphasized the need for “protein and vegetables every night- that’s absolutely.” Many parents were concerned about their children’s health in terms of weight and nutrition. In the case of P14 (male, age 9) who had his colon removed, his father talked about the need to ensure that he had the necessary nutrients, salt and water after his surgery:

Those are the 2 main ones [water and salt]. The water’s the big one. Um, I think the colon, you know, it’s um, yeah, no. Those are the 2 main ones that stand out, those are the two that I really watch the whole time. Otherwise, I, in the back of my mind, I’m under the impression that the, ah, he’s not getting all the nutrients he would normally get. So that’s why I really try to break down the food groups for meals. I really try to make sure that he’s getting all the different food groups in a meal. (F14)

Although participants emphasized healthy eating and consuming foods they considered healthy, it was evident that they were following general notions of healthy eating rather than IBD-specific ones. In some cases, healthy eating was discussed as a part of the home environment even prior to the diagnosis. For others, the diagnosis might have brought about a greater emphasis on healthy eating. While some participants stated that consuming certain foods was good for IBD, a few participants were more uncertain if eating those foods would actually help. When asked if there were any foods that she consumed to help with her symptoms, one participant (P1, female, age 12) said: “Um, not really. I just kinda eat vegetables and fruits. Not really, it doesn’t help.” Her father remarked: “It’s always been easier to point out the foods that were troublesome as opposed to foods that were helping, so I don’t, any food that doesn’t have symptom to me is helping.” (F1)

## Family food practices

### Impact on grocery shopping, meal planning, and cooking

There was much variation in how parents of children with IBD described the impact of IBD on their family food practices. Some of the parents described the impact on grocery shopping, meal planning and cooking as minimal, while others expressed that the illness had a significant impact. Many parents talked about buying foods they considered healthy or good for IBD, as well as avoiding or limiting foods they believed could aggravate symptoms or they considered to be unhealthy. Some parents remarked that IBD had little impact on their family food practices. In the case of P18 (female, age 16), her mother was on a diet recommended by a personal trainer and this had a larger impact than did P18’s condition. P18 was one of the few participants who did not have any trigger foods. Her mother commented that she adapted her diet for her children, even though she prepared a meal for herself and another for her children: “Which is pretty much the same. But like I said, instead of brown rice for them, it’ll be white rice. Um, but to cook the meat, everything else is the same.” (M18)

A few parents mentioned having to juggle the dietary needs of different family members. A father commented that he had to “dance around” his son (P11, male, age 15) having IBD and his daughter being vegetarian and having a peanut allergy among other factors (i.e., costs, nutritional value, preferences). At the same time, he described P11’s condition as not having a great impact on certain household food practices:

In the sense of what comes into the house, uh, I would say very little. We always had a lot of fresh fruit for the kids even before we knew that. Um, I’m a very plain eater, ah, so, that hasn’t really changed, ah so, that hasn’t really changed because, like, there’s nothing more plain than cooking a plain ham. (F11)

For other parents, IBD had resulted in significant changes in their food practices. Some parents mentioned having started cooking from scratch more often or having adapted their ways of preparing food after the diagnosis. Parents might prepare separate dishes for the child with IBD or adopt the changes in diet for the entire family. For example, the father of P14 (male, age 9) mentioned using the slow cooker to make food easier to digest. A small number of parents talked about the impact of IBD on their family’s cultural or traditional ways of eating. This is illustrated in the interview with the mother of P22 (male, age 15) who made changes after finding out corn was a trigger food for her son:

uh, ‘maïs’, um, corn? Yeah, so everything that is corn, we don’t- actually we have- we eat in Eastern Europe ‘polenta’, so we’re not eating any more this. So this is really out of- like anything that has corn we don’t buy. (M22)

In addition, she mentioned trying to cut back on potatoes and buying organic meats whenever possible, as well as incorporating more vegetables in family meals. The cultural/traditional ways of eating were balanced with general notions of healthy eating and a need to cope with and manage IBD symptoms.

#### Maintaining routine and normality

The parents we spoke to tried to maintain a routine of food practices while minimizing the impact of the condition on their family and children. For example, corn and fibrous foods were identified as problematic for P9 (female, age 15) and her brother, P8 (male, age 13). Their father indicated that he and his wife would prepare dishes such that the children could partake in the same meal: “When we make shepherd’s pie we’ll do it with and without corn, right, so they can eat the same thing, or basically the same thing.” (F8.9) In the case of P5 (female, age 14) who had problems with fiber, her mother reported that the family reduced the consumption of pasta and switched to another type of bread when she had active symptoms:

It was easiest for us to just adjust our food for our whole family than to it just for [P5]. That way it wasn’t like [P5] was dealing with this disease on her own. It was more like we were all supporting her and helping her so it would make it easier for her. (M5).

Correspondingly, a few parents expressed a desire to support their children in their dietary needs due to IBD while trying to maintain normality and a routine of food practices for the family. Hall, Rubin, Dougall, Hungin, and Neely (2005) proposed the concept of health-related normality as “that which the participants perceived as usual, ordinary, common or typical in terms of activity, freedom and quality of life” (p. 446). In their study, Hall et al. used this concept to explain the continuous assessment that adults with IBD made regarding their well-being, their fight to maintain normality, and their need to retain the appearance of normality to others. In our interviews, we noted attempts made by the parents so that their children would not feel different or be conscious of having IBD. This is exemplified in the interview with P26’s mother who adopted the SCD in support of her daughter (P26, female, age 10). She described her attempts for the family to have the same or similar meals despite the restrictions imposed by the diet:

I try to make it so- I don’t want [P26] to feel like she has to eat different foods, like that her diet’s totally different, so I will make whatever [P26] and I are having, for everybody and then they may have rice with it or something else, like just, they can have the starches and the other things. So I try and make it similar, so that we’re not eating completely different things. (M26)

At the IBD Clinic, the Dietician recommends restricting the intake of foods that could trigger symptoms during active flare-ups when meeting with patients and their parents. One participant talked about how her family would eat the same foods as she did when she had active symptoms and needed to restrict her intake of foods like red meats:

They usually ate the same things as I did so that I wouldn’t miss, like, ‘oh I miss eating steak,’ or stuff like that. So dad, most of the time he wouldn’t do that. But once in awhile he would have it ‘cause, like, I told him, ‘you can eat it. I’m not gonna cry if you eat steak.’ But most of the time they tried to eat the same things that I would eat so I wouldn’t be like, ‘oh, I miss that.’ (P1, female, age 12)

Many parents mentioned talking to their children about IBD and food. P25 (female, age 14) currently did not have any food restriction although she generally avoided foods high in sodium or fat. When asked if he talked to her about food and nutrition, her father said:

Yes, of course, and especially when she was younger, um, and she had a nutritionist [i.e., Dietician] at [the clinic] who also talked to her about it, so yeah, oh, very much so. Yeah she knows- oh yeah we were open and we talked openly about it and, because it’s- there’s nothing to be embarrassed about and there’s nothing wrong. We never made the- we never made a big deal about it. You have this inflammation and we’re gonna get it under control and I want you to eat as- but- as- as well as you can and you’re gonna be fine and- she doesn’t even- she doesn’t even realize she has it anymore, I don’t think. (F25)

He further commented that P25 had taken her medication for 11 years and that “it’s just part of her- it’s like brushing her teeth.” His comments demonstrated the adaptation that occurred as pediatric patients and their parents adjusted to living with IBD. Likewise, Hall et al. (2005) found that adults with IBD described their illness as something that they had learned to live with and had become a normal part of life. Cooper, Collier, James, and Hawkey (2010) also found that adult patients with IBD expressed a resolve to make their illness a part of normal life.

## Discussion

The purpose of this study was to explore the daily food experiences of pediatric patients and parents living with IBD. The participants had diverse experiences and perspectives about the role of food and diet. Most of them, including both the pediatric patients and their parents, took an active role in managing IBD through their food practices. It has been reported that adult patients with IBD viewed having a degree of personal control as important, although this involved having to adapt to the uncertain and unpredictable nature of the illness (Cooper et al., 2010). We identified three types of dietary strategies that pediatric patients and their parents used to help manage IBD symptoms, which might be seen as ways to strive for personal control over the illness. It is not clear from the literature how widespread dietician consultation is part of routine IBD care in adult or pediatric IBD clinics; but it is the standard of care for the IBD Clinic we recruited participants from for this study and so may have a confounding effect on participants’ perceived control over the illness and their responses during the interview. The three types of dietary strategies we identified included food avoidance and moderation, following a specific diet, and healthy eating. These strategies were not mutually exclusive and were often used in conjunction with each other in an effort to manage the illness. Many participants who emphasized healthy eating also avoided or moderated the intake of foods they considered to be trigger foods. Conversely, those who avoided certain trigger foods often emphasized healthy eating.

The findings of this study lend support to previous research on the relationship between diet and IBD among adult patients. In particular, avoidance of and challenges to abstaining from trigger foods have been documented among adult patients with IBD (Fletcher & Schneider, 2006; Palant et al., 2015; Schneider et al., 2009). Fletcher and Schneider (2006) also briefly reported on healthy eating among their participants. In their study, healthy eating was described as consuming “items that were healthier options” and “items that would help with digestion or the condition itself” (p. 245). The same aspects of healthy eating were observed in this study, which provided a more detailed analysis on what healthy eating meant for pediatric patients with IBD and their parents. Many participants discussed healthy eating as being beneficial for IBD, either to help them manage the symptoms or to improve health physically and mentally. For the majority of these participants, healthy eating consisted of consuming more fruits and vegetables and avoiding foods that were perceived as unhealthy (e.g., foods high in sugar, fast foods). The Pediatric IBD Porto Group from ESPGHAN recommends avoidance of a “westernized” diet that is high-fat, high-protein, high-sugar, and low in fruits and vegetables since it has been identified as risk factor for the development of IBD in several epidemiological studies (Miele et al., 2018).

On a practical level, these findings have important implications for the clinical care of pediatric IBD. First, our analysis shows that the impact of IBD on food practices is not limited to the individual, but extends to the family. Healthcare professionals including physicians, nurses, and dieticians may want to consider the family unit when giving dietary recommendations or developing nutritional guidelines. Dietary interventions should also be more family oriented. Parents and other family members may follow the same diet as the patient as it is difficult to prepare separate meals. Thus, they need to know about the potential impact of following such a diet on themselves in terms of nutrition. In this study, the impact of IBD on family food practices, or lack thereof, was not necessarily related to symptom severity. Other factors, such as family food practices prior to the diagnosis and dietary needs of different family members, also had significant influences. It is important for healthcare professionals to recognize the desire of parents to maintain a routine of family food practices and normalize the impact of IBD on their children. Healthcare professionals can assist patients and families in identifying trigger foods and making decisions surrounding healthy eating in their daily lives without significant disruptions to the children and other family members. Nutritional recommendations may also be more likely to be adhered to if they can be made to fit into family routines and practices.

Secondly, the relationship between IBD and food is dynamic. In this study, participants explained that what they or their children ate or avoided to eat depended on whether they were in remission or having active flare-up. This is reinforced by the IBD Clinic Dietician during interactions with patients and their families, along with the need for ongoing monitoring and reassessment of nutritional needs. It has been noted that dietary restriction can increase the risk of deficiencies in key nutrients, highlighting the need for nutritional assessment and screening for growth impairment in pediatric patients (Kleinman et al., 2004; Lucendo & De Rezende, 2009; Massironi et al., 2013). A reduced consumption of trigger foods that are important sources of nutrients should be carefully monitored and substitutes may need to be identified for key foods. The Pediatric IBD Porto Group from ESPGHAN recommends assessment of dietary intake at least twice per year in small children aged 5 or younger and once per year in older children and adolescents, or whenever deemed necessary by the treating physician and/or dietician (Miele et al., 2018). Since IBD is known to be associated with delayed pubertal development, regular assessment of pubertal stage is also recommended for children aged 10 years and older, and at least annually during follow-up visits until puberty is completed (Miele et al., 2018). Given the heterogeneity of patient conditions and familial circumstances, personalized nutritional counselling is highly recommended for pediatric patients and their families.

There are several limitations to this study. Our sample was selected from a clinic at a major children’s hospital in Ontario and might not be representative of the pediatric IBD population as a whole. Moreover, the clinic is located in a major metropolitan area with high median household income compared to other regions of Canada (Statistics Canada, 2017), which may have an impact on the participants’ food strategies given the costs of food. Research with samples from other clinical or sociocultural contexts may reveal different experiences and perspectives. Additionally, there were few younger children at or below the age of 12 in our sample (*n* = 6). Recruiting a larger sample of younger children may provide insights into the food experiences of pediatric patients during different stages of development. This study focused on the impact of IBD on certain household food practices such as grocery shopping, meal planning and cooking. Future research should explore the effects of IBD on other aspects of the food experiences of pediatric patients and their parents, such as eating out or attending social events. Individuals with IBD, including children and adolescents, often face challenges associated with managing their condition in social situations. Among adults, concerns about food consumption away from home, such as when travelling, eating out at a restaurant or attending a social event, have been documented in several studies (Fletcher & Schneider, 2006; Kinsey & Burden, 2016; Palant et al., 2016; Prince et al., 2011; Schneider et al., 2009; Zallot et al., 2013). Understanding how pediatric patients deal with food consumption in different social situations can help inform clinical care and nutritional counselling. More research is also needed to understand the importance of food strategies as a means to cope with IBD for pediatric patients and their families.

## Conclusions

To our knowledge, this is the first qualitative study to explore the food practices of pediatric patients with IBD who were not on a dietary intervention and their families. Most of the participants, including children and adolescents and their parents, took an active role in their management of IBD symptoms through daily food practices. We identified three types of dietary strategies that participants used, including food avoidance and moderation, following a specific diet, and healthy eating. Our findings highlight the need for healthcare professionals to consider the family unit when giving nutritional advice or developing nutritional guidelines. Personalized nutritional counselling and ongoing nutritional assessment are also warranted.
